# A machine learning approach of predicting high potential archers by means of physical fitness indicators

**DOI:** 10.1371/journal.pone.0209638

**Published:** 2019-01-03

**Authors:** Rabiu Muazu Musa, Anwar P. P. Abdul Majeed, Zahari Taha, Siow Wee Chang, Ahmad Fakhri Ab. Nasir, Mohamad Razali Abdullah

**Affiliations:** 1 Innovative Manufacturing, Mechatronics and Sports Laboratory, Faculty of Manufacturing Engineering, Universiti Malaysia Pahang, Pekan, Malaysia; 2 Faculty of Applied Social Sciences, Universiti Sultan Zainal Abidin, Kuala Terengganu, Malaysia; 3 Bioinformatics Programme, Institute of Biological Sciences, Faculty of Science, University of Malaya, Kuala Lumpur, Malaysia; University of Illinois at Urbana-Champaign, UNITED STATES

## Abstract

*k*-nearest neighbour (*k*-NN) has been shown to be an effective learning algorithm for classification and prediction. However, the application of *k*-NN for prediction and classification in specific sport is still in its infancy. The present study classified and predicted high and low potential archers from a set of physical fitness variables trained on a variation of *k*-NN algorithms and logistic regression. 50 youth archers with the mean age and standard deviation of (17.0 ± 0.56) years drawn from various archery programmes completed a one end archery shooting score test. Standard fitness measurements of the handgrip, vertical jump, standing broad jump, static balance, upper muscle strength and the core muscle strength were conducted. Multiple linear regression was utilised to ascertain the significant variables that affect the shooting score. It was demonstrated from the analysis that core muscle strength and vertical jump were statistically significant. Hierarchical agglomerative cluster analysis (HACA) was used to cluster the archers based on the significant variables identified. *k*-NN model variations, i.e., fine, medium, coarse, cosine, cubic and weighted functions as well as logistic regression, were trained based on the significant performance variables. The HACA clustered the archers into high potential archers (HPA) and low potential archers (LPA). The weighted *k*-NN outperformed all the tested models at itdemonstrated reasonably good classification on the evaluated indicators with an accuracy of 82.5 ± 4.75% for the prediction of the HPA and the LPA. Moreover, the performance of the classifiers was further investigated against fresh data, which also indicates the efficacy of the weighted *k*-NN model. These findings could be valuable to coaches and sports managers to recognise high potential archers from a combination of the selected few physical fitness performance indicators identified which would subsequently save cost, time and energy for a talent identification programme.

## Introduction

The sport of archery is regarded as a fine and gross motor skilled based sport. Elite and highly skilled archers are distinguished by their abilities to shoot at a target constantly within the stipulated time with a high level of accuracy. A recent study has provided evidence supporting that to attain mastery in archery sport, a significant level of physical fitness is necessary [1]. There is also the existence of literature underlining the relevance of physical fitness indicators such as core body strength, upper body strength, handgrip, leg power and static balance to the achievement of high archery scores [2,3]. Similarly, it has been reported that the performance level of an archer could improve significantly when all the necessary physical fitness components are acquired [4]. The authors stressed that the sport comprises of some distinct aerobic and anaerobic activities and as such, the major muscle fibres are activated during the stance, aiming, and the releasing of the arrow. Moreover, Spencer et al. documented that physical fitness components are essential for successful performance in the sport of archery [5].

The employment of machine learning or artificial intelligence has gained popularity in predicting and classifying physiological properties as well as activity type owing to its superiority over conventional means [6]. Artificial Neural Networks (ANN) was used to predict energy expenditure (EE) from body-worn accelerometers attached to the right hip, right thigh as well as both wrists [7]. The accuracy of the ANN prediction was compared to linear regression as well as linear mixed models, and it was conclusive from the study that the ANN model is superior in providing a better prediction accuracy in comparison to the other conventional evaluated models.

The application of ANN has also been investigated in predicting activity type of preschool children [8]. Five categories of activity classes were classified namely, sedentary, moderate to vigorous, light, walking and running by placing an accelerometer on the right hip of eleven children between the age of three and six years old. Two different neural network algorithms were compared namely conventional feed-forward Multi-Layer Perceptron (MLP) ANN and deep learning ensemble network (DLEN). It was found in their study that the DLEN provided a better overall recognition accuracy of 82.6% whilst the MLP ANN, 69.7%.

The prediction of EE, as well as the classification of physical activity (PA), type viz. household, stairs, walking and running by considering heart rate (HR) data, as well as accelerometer data obtained from the wrist and hip through the employment of random forest (RF) classifier, was carried out by Ellis et al. [9]. Furthermore, they employed random forest regression trees to estimate metabolic equivalents (MET). The study indicates that both random forest classification, as well as regression forest, was able to classify well the PA type as well as predicting MET, respectively.

The classification of seven distinct activity classes namely lying down, sitting, standing, walking, running, basketball as well as dancing through acceleration signals obtained from the wrist and hip by means of regularised logistic regression was investigated by Trost et al. [10]. A three-fold cross-validation technique was employed to evaluate the predictability of the machine learning model developed. It was established that the machine learning model driven by the wrist and/or hip acceleration data could classify the aforementioned PAs well.

In a recent study, Pavey et al. also utilised RF classifier to classify different forms of activity (sedentary, stationary+, walking and running) through data obtained via wrist-worn accelerometer [11]. The leave-one-out-cross-validation method was used to assess the model performance, and it was shown that the RF algorithm provided an overall classification accuracy of 92.7%, suggesting the efficacy of such machine learning algorithm to classify PA.

Furthermore, RF has been used to classify the playing position of elite junior Australian football based on technical skill indicators in addition to linear discriminant analysis as well as PART decision list [12]. A fivefold cross-validation was used to evaluate the models. It was demonstrated from the study that the PART decision list provided the best classification accuracy amongst other models, in classifying midfielders, ruckmen, and defenders, respectively. Nonetheless, it was also shown that accurate classification solely based on technical skills is unattainable.

It is apparent from the literature that the employment of machine learning is a useful tool for prediction as well as classification. The *k*-Nearest Neighbour (*k*-NN) is a non-parametric regression and classification method developed by Fix and Hodges in the 1950s [13]. It is regarded as one of the simplest forms of supervised machine learning algorithms. Nonetheless, *k*-NN did not gain considerable attention until the sixties owing to the limited computing power prior to it. This primitive form of machine learning is also known as ‘lazy learning’ or ‘instance-based learning ‘as it does not require learning, in other words, the computation of the algorithm transpires during runtime [14]. *k*-NN has been successfully employed for classification in a number of different fields [15–19].

Hitherto, to the best of the authors’ knowledge, the application of *k*-NN to classify potential athletes in the sport of archery has not yet been investigated. This study aims at correlating the selected physical and motor ability performance variables, i.e., hand grip, vertical jump, standing broad jump, static balance, upper muscle strength and core muscle strength, respectively in identifying future potential archers. It is worth mentioning that prior to the cluster analysis, the multiple linear regression is applied to identify the significant performance indicators that influence the shooting score.The hierarchical agglomerative cluster analysis (HACA) is employed to cluster the archers based on their performances in the identified variables and their archery shooting scores in which two classes, i.e., high potential archers (HPA) and low potential archers (LPA) are clustered. Moreover, the performance of the variation of *k*-NN in classifying HPA as well as LPA is examined against the conventional logistic regression classifier.

## Materials and methods

### Participants

A total of 50 archers were recruited to take part in this study. The participants comprised of 37 male and 13 female youth archers in the age’s range of 13 to 20 with a mean and standard deviation of (17.0 ± 0.56) years assembled from varying archery programmes in Malaysia. The archers were under a development program for preparing both at university and the state level and as a result, targeted to represent state and national archery competitions. Normality assessment was run using Shapiro-Wilk, and the archers were found to be equivalently distributed. The coaches and the stakeholders of the council were notified about the aim of the study. Written consent was obtained, and all the archers signed consent forms. All the procedures, protocol, and equipment for this study were reviewed and authorised by the Research Ethics Board of the Terengganu Sports Institute (ISNT) with an approval number of 04-04/T-01/Jid 2. Moreover, consent from the parents/guardians of the participants who are under the age of 18 years old was obtained through Universiti Sultan Zainal Abidin’s Ethical Committee for Human Research (UHREC/2017/2/003).

### Physical fitness assessment

Standard physical fitness indicators of sit up, push up, standing stork test, handgrip test, standing broad jump test and vertical jump test was conducted in accordance with standard physical fitness assessments [20–22]. The participants performed a warm-up that comprised of a 5- to 10-min jog and a series of stretches prior to the testing sessions. The push-up and sit up tests were performed using a mat spread on the floor which measures the upper muscle strength (UMS) and core muscle strength (CMS), respectively. The archers completed the tests alternately under a period of 1-minute once for each test. The standing stork (SS) test was conducted using a stopwatch. The archers removed their shoes and placed their hands on their hips and raised their heel to balance on the ball of the foot then positioned the non-supporting foot against the inner part of the supporting leg. The test is stopped in the event that the archers could no longer maintain the exact position. The hand grip (HG) strength of both hands was measured using a standard adjustable grip strength dynamometer (Takei Scientific Instruments Co., LTD). The archers were instructed to stand in an upright position with the shoulder in adduction and neutral rotation and elbow in full extension. The standing broad jump (SBJ) was examined using a landing mat placed on the flat synthetic surface, and the take-off line clearly indicated from behind the mat. A two-foot take-off and landing are utilised, with the swinging of the arms and bending of the knees to provide forward drive. Three attempts were allowed, and the best was used for the statistical analysis. The vertical jump (VJ) was assessed using Vertec (M-F Athletic Co., Cranston, Rhode Island). The researchers adjusted the height of the colour-coded plastic vanes such that it paralleled to the archer's standing height. The archers flexed the ankles, knees, and hips and swung the arms in an upward motion whilst tapping the highest possible vane with the fingers of their dominant hand. The best of the three trials was utilised for statistical analysis. The archery shooting (AS) test was implemented prior to the aforementioned fitness testing using a replicated competition shooting area of 50 meters distance. The archers were given four trial shots, before recording the final six arrows scores.

### Feature extraction and cluster analysis

Multiple linear regression (MLR) was employed in order to facilitate the determination of the variables that significantly influence the archery shooting score amongst the evaluated variables. The archery shooting score was assigned as the dependent variable, whilst the physical fitness variables are treated as the independent variables. In the current study, hierarchical agglomerative cluster analysis (HACA) was used to separate or cluster the classes of the related performance variables assessed using XLSTAT 2014 add-in software.

### Classification

The supervised learning for classification, on the other hand, is carried out by means of a variation of *k*-NN and the conventional logistic regression classifiers. The concept of *k*-NN is fairly straightforward in which a sample is assigned to a predefined class per the majority of its *k*-nearest neighbour in the data space. The distance metric is then employed to compute the distance between the individual samples from all the other samples prior to being sorted based on the distance. In this study, six variations of *k*-NN are investigated namely, fine, medium, coarse, cosine, cubic and weighted.

The number of neighbours, *k* for fine and coarse are 1, and 100 whilst the remaining variation, i.e., medium, coarse, cosine, cubic and weighted, the number of neighbours are selected to be 10. As for the distance metrics, Euclidean distance is utilised in the fine, medium, coarse and weighted *k*-NN variations, whilst the cosine and cubic *k*-NN variations employ the cosine and a special case of the Minkowski distance, respectively. The ‘no distance weight’ (i.e., equal distance) was used for all variations except the weighted k-NN, in which, the weight is the squared inverse of the Euclidean distance. The distance formulae for Euclidean (*d*_*e*_), cosine (*d*_*c*_) and cubic (*d*_3_) are as follows:

$$d_{e}\left(x_{i},x_{j}\right)=\sqrt{\sum_{r=1}^{n}{\left|\left(x_{i}\right)-\left(x_{j}\right)\right|}^{2}}$$ (1)

$$d_{c}\left(x_{i},x_{j}\right)=1-\frac{x_{i}.x_{j}}{\sqrt[{}]{\left(x_{i}.x_{i}\right).\left(x_{j}.x_{j}\right)}}$$ (2)

$$d_{3}\left(x_{i},x_{j}\right)=\sqrt[{3}]{\sum_{r=1}^{n}{\left|\left(x_{i}\right)-\left(x_{j}\right)\right|}^{3}}$$ (3)

Where *d* is the distance between new data point *x*_*i*_ and training data point *x*_*j*_

Logistic regression is a statistical modelling method that is based on the notion that a sigmoidal relationship exists between the probability of group membership as well as the predictor variables [23–26]. The logistic model may be represented via the following equations:

$$z=\beta_{0}+\sum_{i=1}^{n}\beta_{i}x_{i}$$ (4)

$$P\left(1|z\right)=\frac{e^{z}}{1+e^{z}}$$ (5)

where *z* is a measure of the influence of the descriptive variables *x*_*i*_ (*i* = 1, …, *n*), *β*_*i*_ are the regression coefficients which are obtained by maximum likelihood in conjunction with their standard errors Δ*β*_*i*_, and *P*(*z*) is the categorical response of the variables that represent the probability of an archer to be categorised as HPA or LPA.

A fivefold cross-validation technique was utilised in this study as it mitigates the notion of overfitting through partitioning the dataset into a number of folds and estimating the accuracy of each fold. 40 observations are randomly split into five subsets, and for each iteration, one of the 5 subsets is used as the testing data, whilst the remaining four will be used as the training data. Then, the average performance over all the folds is then computed. The performance of the *k*-NN models was assessed and evaluated via MATLAB 2016a (Mathworks Inc., Natick, USA). In addition, ten fresh data (that was not used in training the models) is fed into the developed models to evaluate its efficacy in classifying the performance classes of the archers, i.e., HPA and LPA.

The variations of the *k*-NN models, as well as the logistic regression classifier employed in this study, are evaluated by means of classification accuracy (ACC), specificity (SPEC), precision (PREC), sensitivity (SENS), error rate (ERR), as well as Matthew’s correlation coefficient (MCC). The ACC is essentially the ratio between the number of correctly classified observations and the total number of observations. The SENS (also known as recall) and the SPEC are the true positive rate or the positive class accuracy as well as the true negative rate or negative class accuracy, respectively. The PREC computes the number of correct positive predictions over the total number of positive predictions. The ERR, on the other hand, appraises all misclassifications over the number of total observations. Conversely, the MCC is a discrete version of the Pearson’s correlation coefficient that measures the quality of binary classification, and it has a range of -1 to 1 whereby 1 suggests a completely correct binary classifier and -1 suggests otherwise. It gauges the performance of the classification models.

The confusion matrix allows the observation of correctly classified and misclassified observations that transpires between the defined classes. The detailed method of acquiring the aforementioned assessment parameters are given in the Supporting information section.

## Results and discussion

Table 1 shows the regression analysis carried out to determine the association of the fitness variables with the archery shooting score. It can be observed from the table that only VJ and CMS are significantly associated with the archery shooting score with relatively higher contribution from the standardised beta coefficient. Based on the outcome of this analysis, therefore, only the significant variables are considered for the clustering, i.e. AS, VJ and CMS.

**Table 1:** Multiple linear regression analysis for feature extraction.

Variables	Std. Error	Beta	t	Sig
**AS**	8.167	-	5.765	.001
**VJ**	.168	.391	2.464	**.018**
**SBJ**	.048	.313	1.557	.127
**SS**	.019	-.031	-.199	.843
**HG**	.101	-.144	-.856	.397
**UMS**	.128	-.131	-.794	.431
**CMS**	.228	.299	1.726	**.026**

**Fig 1:**
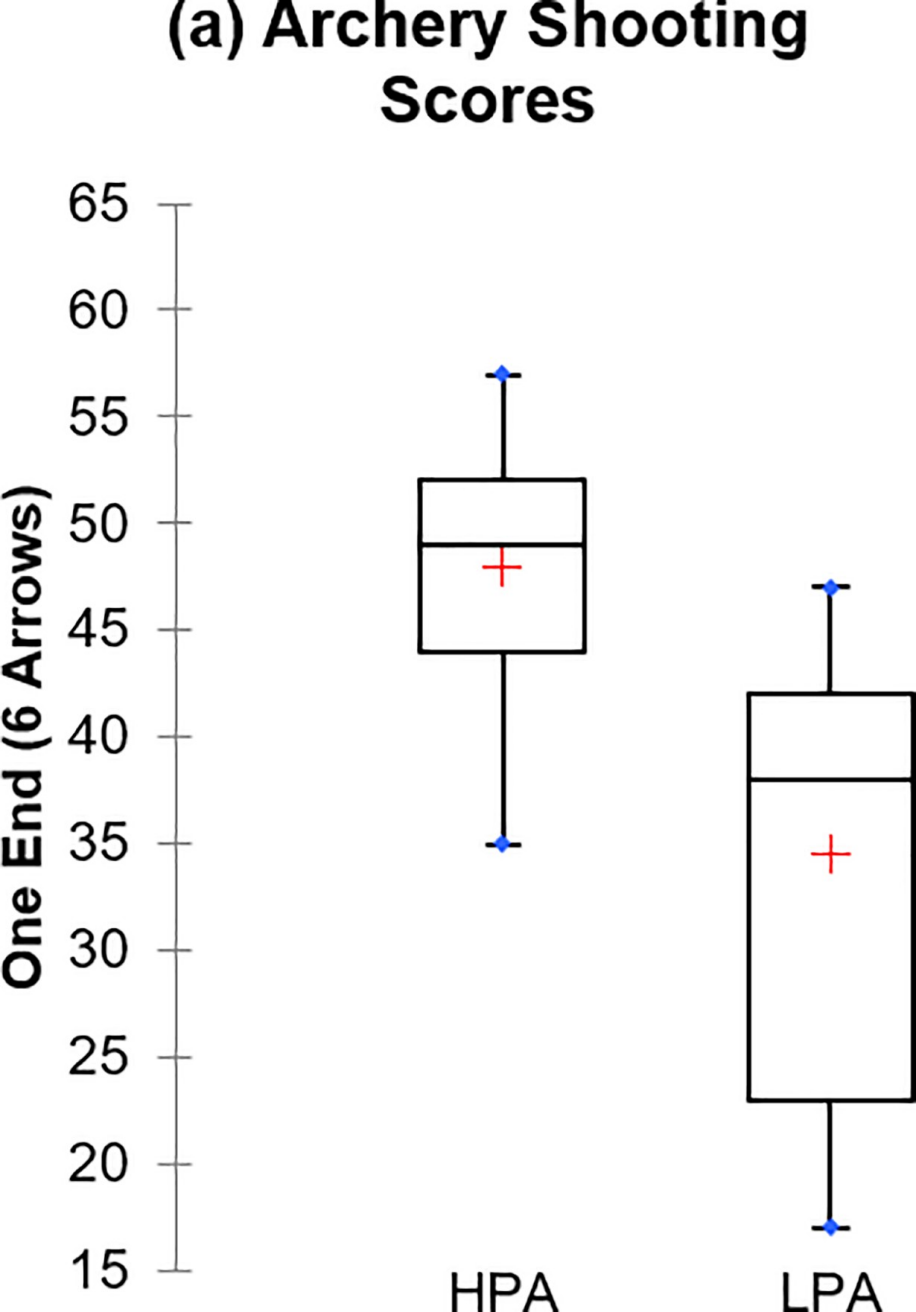
Box plots of the significant variables evaluated. (a) Archery shooting score.; (b) Vertical Jump; (c) Core Muscle Strength.

Table 2 indicates the inferential analysis carried out to determine the difference in performance between HPA and LPA with respect to the identified significant variables. It could be seen from the table that there is a statistically significant difference between the group with regards to AS, as well as VJ (p < 0.001). However, no significant difference is observed between the group for CMS (p > 0.05) which indicates that both the HPA and LPA could not be distinguished solely based on the CMS. Nonetheless, it is worth to note at this juncture that CMS could not be omitted in the subsequent analysis, i.e., classification, as it was found to be significant in influencing the archery shooting score as demonstrated via the MLR analysis. Fig 1 displays the performance differences of the archers based on the significant variables identified. It can be observed from the box plots that the mean performances of HPA are greater than LPA across all the identified variables measured in the study. These variables are, therefore, considered as essential attributes that distinguish HPA from LPA.

**Table 2:** The inferential analysis of the pairwise comparison between the HPA and LPA.

Variables	t	DF	Mean ± SD	Sig
**AS**	5.870	48	47.95±5.10.58	**.0001**
**VJ**	5.725	48	38.71±8.64	**.0001**
**CMS**	0.307	48	29.62±5.43	.760

**Fig 2:**
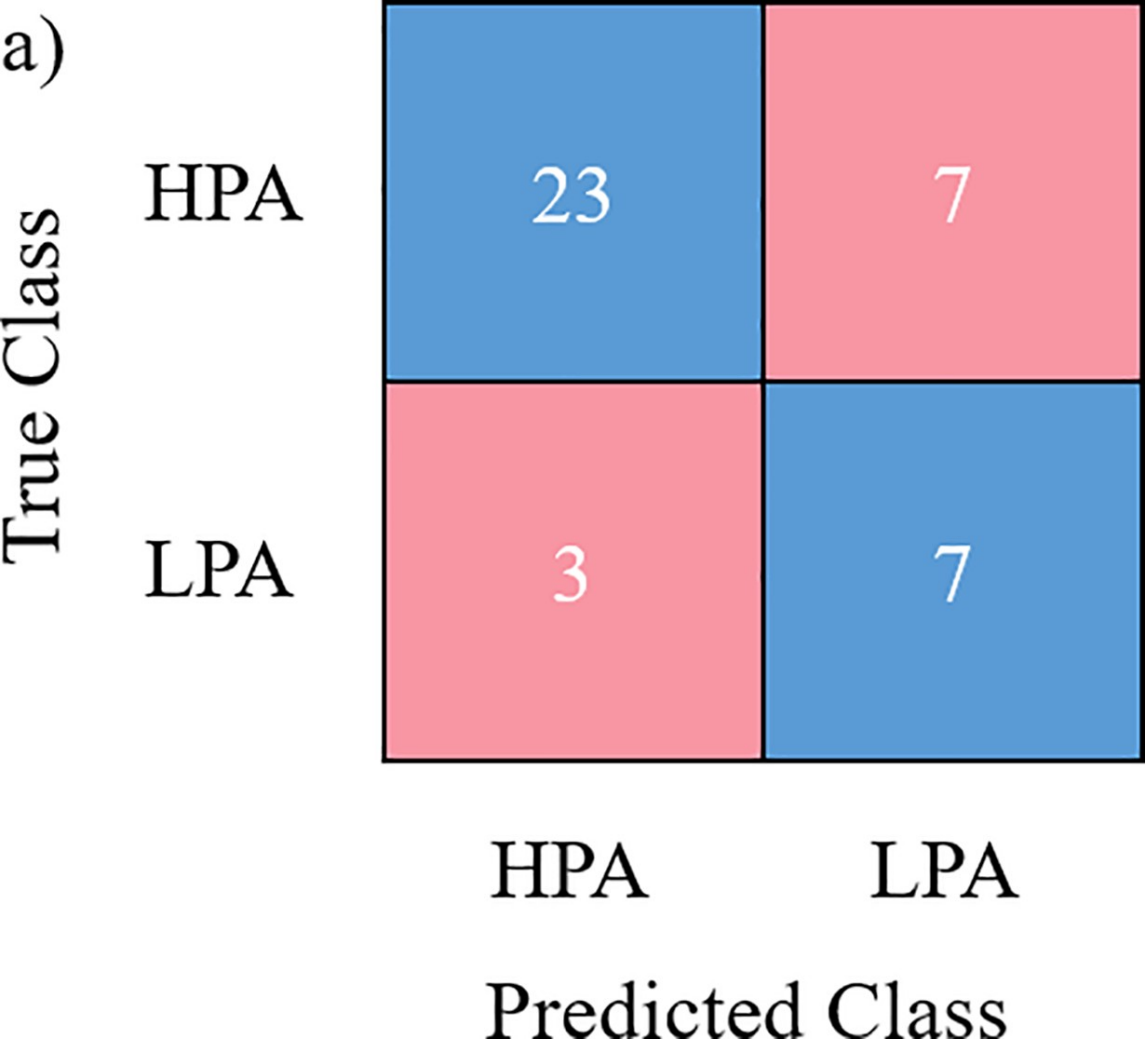
Confusion matrix. (a) Fine; (b) Medium; (c) Coarse; (d) Cosine; (e) Cubic; (f) Weighted; (g) Logistic Regression.

Table 3 tabulates the performance of the evaluated variation of *k*-NN algorithms employed as well as the logistic regression algorithm. It is evident that the weighted *k*-NN variation outperforms other classifiers evaluated. Moreover, it could also be observed through the MCC metric, that the weighted *k*-NN variation has a better correlation amongst the other evaluated classifiers. The logistic regression model yields the second highest classification accuracy with a classification accuracy of 80%. This is followed by the cosine *k*-NN variation. It was demonstrated in terms of classification accuracy that the coarse, fine and cubic variation of the *k*-NN models performs equally. It is worth noting that the lower the *k*-value, the decision boundary tends to have a more flexible fit with less bias but at the expense of a higher variance. This observation is apparent for the fine variation as it has both reasonably high sensitivity. As the *k* grows, the less susceptible it is towards outliers as a smoother decision boundary is formed with lower variance but with an increased level of biasness. This is prevalent with the selection of *k* = 10 as the specificity is much lower than of the fine *k*-NN variation. The variation of the distance metric employed for those of which *k* = 10 models further affect the classification accuracy of the models, and this is noticeable through the higher classification accuracy and acceptable specificity attained by the weighted *k*-NN variation. It is also apparent from the table that by far the worst classifier is the medium *k*-NN variation. It is worth noting that the decision boundary provided by the logistic regression classifier tends to be linear and is not as robust as *k*-NN classifiers that are able to cater non-linear boundaries. This further suggests the ability of the weighted *k*-NN classifier to outperform the logistic regression classifier. Fig 2 depicts the performance of the confusion matrix of the respective variation of the *k*-NN algorithm as well as the logistic regression.

**Table 3:** The performance evaluation of the variation of *k*-NN algorithms and logistic regression.

Algorithms	ACC (%)	SENS (%)	SPEC (%)	PREC (%)	ERR (%)	MCC
**Fine** ***k*-NN**	75.00± 5.10	88.46	50.00	76.67	25.00	0.4237
**Medium** ***k*-NN**	72.50 ± 4.30	74.36	0.00	96.67	27.50	-0.0925
**Coarse** ***k*-NN**	75.00 ± 4.55	75.00	NA	100.00	25.00	NA
**Cosine** ***k*-NN**	77.50 ± 6.25	78.38	66.67	96.67	22.50	0.2740
**Cubic** ***k*-NN**	75.00 ± 5.90	76.32	50.00	96.67	25.00	0.1325
**Weighted *k*-NN**	82.50 ± 4.75	71.43	93.33	50.00	17.50	0.4938
**Logistic Regression**	80.00 ± 5.45	82.35	66.67	93.33	20.00	0.4042

Table 4 illustrates the prediction efficacy of the developed models in classifying HPA and LPA against fresh data. This additional analysis is non-trivial in order to investigate the efficacy of the classifiers against unseen data to further validate the models developed. It could be observed from the table that the weighted *k*-NN classifier misclassified only one athlete from the LPA group (the bold fonts indicate the misclassified class). Moreover, it is evident that the logistic regression trained model misclassified three archers from each group. Based on this supplementary analysis, it could be concluded that the best model to discriminate the performance classes of the archers is the weighted variation of the *k*-NN model.

**Table 4:** Classification efficacy of the developed models against fresh data.

	Classification Prediction						
Actual Classification	Fine	Medium	Coarse	Cosine	Cubic	Weighted	Logistic Regression
LPA	**HPA**	**HPA**	**HPA**	LPA	**HPA**	LPA	**HPA**
LPA	LPA	**HPA**	**HPA**	**HPA**	**HPA**	LPA	LPA
LPA	**HPA**	**HPA**	**HPA**	**HPA**	**HPA**	**HPA**	**HPA**
HPA	HPA	HPA	HPA	HPA	HPA	HPA	HPA
HPA	HPA	HPA	HPA	HPA	HPA	HPA	HPA
HPA	HPA	HPA	HPA	HPA	HPA	HPA	**LPA**
HPA	HPA	HPA	HPA	HPA	HPA	HPA	HPA
HPA	HPA	HPA	HPA	HPA	HPA	HPA	HPA
HPA	HPA	HPA	HPA	HPA	HPA	HPA	HPA

The aim of the study is to investigate the capability of the variation of the *k*-NN classifier against the logistic regression classifier in identifying either high or low-potential archers. The shooting scores enable us to cluster the athletes in respect to identified significant physical fitness tested namely vertical jump, and the core muscle strength via HACA as illustrated in Fig 1. The performance of the *k*-NN models, as well as the conventional logistic regression classifier, are then investigated in terms of its efficacy in classifying the categories of the archers correctly, and it was demonstrated that the weighted variation of the *k*-NN algorithm exhibits reasonably high ACC, SENS, and MCC, suggesting its capability to accurately classify the performance of the archers to a certain degree of confidence as shown in Table 3. Furthermore, the supplementary analysis carried out by utilising fresh data further validates the classification efficacy of the model. In other words, the identified performance variables are vital in the sport of archery as based on solely on these parameters, a reasonably accurate classification was established. The importance of the selected variables has been reported in the literature as a prerequisite for a better archery performance evaluation.

Landers et al. supported that archery is a sport that requires endurance and muscular strength to cope with the fitness demand for the sport as it involves constant shooting and retrieving the arrows shot [27]. Therefore, muscular strength and endurance would provide considerable benefits to the archers to perform better. According to Keast and Elliott archery requires core muscle strength which actuates the essential muscle groups [28]. The capacity of the essential muscle groups to react to the command called upon them during the performance of the sport enable the archers to shoot the arrow to the point efficiently. It has furthermore been indicated that leg power which is a direct consequence of the ability of the archer to execute a vertical jump, in turn, does reflect the ability of the archers to stand steadily and continuously throughout the shooting period [29,30].

Furthermore, Musa et al. deduced that the combination of leg power and core muscle strength components are essential for providing stability, that in turn influences the arrow target accuracy [1]. The superior stability of the archer will lead to a smaller angle of the parabolic trajectory of the arrow. This will minimise any obstruction from the impacts of the extraneous factors which could change the course of the arrow. Therefore, based on this clarification it can be understood that there is a relatively strong correlation between core muscular strength, and leg power towards the attainment of high performance in archery. The capacity of the archer to utilise the aforesaid performance indicators play a substantial role in determining their performance.

## Conclusions

The current study has illustrated that the essential physical fitness indicators identified, i.e., vertical jump, and the core muscle strength does influence on the determination of the archers’ performance quality. The study also has demonstrated that the use of machine learning algorithms, in particular, the ability of *k*-NN algorithms to reasonably forecast the class of the archers in relation to the identified performance indicators. The resulting *k*-NN variation viz. fine, medium, and weighted, as well as the logistic regression classifiers with the exception of the cosine and cubic *k*-NN has presented reasonable accuracy and precision with a less misclassification rate. Moreover, the weighted *k*-NN model appears to be the best model amongst the selected models throughout the exercise. It is, thus, reliable to presume that the use of such machine learning method is non-trivial as it allows the researchers to correctly identify high potential athletes in the sport of archery by considering few important physical fitness related performance indicators. Future study should consider other related performance indicators associated with the sport as well as other non-conventional classification methods.

### Practical implications

A combination of the selected few identified physical fitness performance indicators is able to recognise high potential athletes. The weighted *k*-NN machine learning algorithm is able to provide reasonably classification of the potential class of the archers. The machine learning approach adopted in this study serves useful for coaches and trainers in identifying potential talent in the sport of archery with relatively fewer related performance indicators.

## Supporting information

S1 File Classification assessment formulae. (DOCX)

S1 Table Raw data. (XLSX)

## References

[1] Musa RM, Abdullah MR, Maliki, Kosni NA, Haque M. The Application of Principal Components Analysis to Recognize Essential Physical Fitness Components among Youth Development Archers of Terengganu, Malaysia. Indian J Sci Technol. Indian Journal of Science and Technology; 2016;9: 1–6.

[2] Martin PE, Siler WL, Hoffman D. Electromyographic analysis of bow string release in highly skilled archers. J Sports Sci. Taylor & Francis; 1990;8: 215–221. 10.1080/02640419008732147 2084268

[3] Ertan H, Kentel B, Tümer ST, Korkusuz F. Activation patterns in forearm muscles during archery shooting. Hum Mov Sci. 2003;22: 37–45. Available: http://www.ncbi.nlm.nih.gov/pubmed/12623179 12623179 10.1016/s0167-9457(02)00176-8

[4] Soylu AR, Ertan H, Korkusuz F. Archery performance level and repeatability of event-related EMG. Hum Mov Sci. Elsevier; 2006;25: 767–774. 10.1016/j.humov.2006.05.002 16859789

[5] Spencer M, Lawrence S, Rechichi C, Bishop D, Dawson B, Goodman C. Time–motion analysis of elite field hockey, with special reference to repeated-sprint activity. J Sports Sci. Taylor & Francis Ltd; 2004;22: 843–850. 10.1080/02640410410001716715 15513278

[6] Robertson S. Improving load/injury predictive modelling in sport: The role of data analytics. J Sci Med Sport. Elsevier; 2017;18: e25–e26. 10.1016/j.jsams.2014.11.198

[7] Montoye AHK, Begum M, Henning Z, Pfeiffer KA. Comparison of linear and non-linear models for predicting energy expenditure from raw accelerometer data. Physiol Meas. IOP Publishing; 2017;38: 343–357. 10.1088/1361-6579/38/2/343 28107205

[8] Hagenbuchner M, Cliff DP, Trost SG, Van Tuc N, Peoples GE. Prediction of activity type in preschool children using machine learning techniques. J Sci Med Sport. 2015;18: 426–431. 10.1016/j.jsams.2014.06.003 25088983

[9] Ellis K, Kerr J, Godbole S, Lanckriet G, Wing D, Marshall S. A random forest classifier for the prediction of energy expenditure and type of physical activity from wrist and hip accelerometers. Physiol Meas. 2014;35: 2191–2203. 10.1088/0967-3334/35/11/2191 25340969 PMC4374571

[10] Trost SG, Zheng Y, Wong W-K. Machine learning for activity recognition: hip versus wrist data. Physiol Meas. 2014;35: 2183–2189. 10.1088/0967-3334/35/11/2183 25340887

[11] Pavey TG, Gilson ND, Gomersall SR, Clark B, Trost SG. Field evaluation of a random forest activity classifier for wrist-worn accelerometer data. J Sci Med Sport. 2017;20: 75–80. 10.1016/j.jsams.2016.06.003 27372275

[12] Woods CT, Veale J, Fransen J, Robertson S, Collier NF. Classification of playing position in elite junior Australian football using technical skill indicators. J Sports Sci. Routledge; 2017; 1–7. 10.1080/02640414.2017.1282621 28125339

[13] Han J, Pei J, Kamber M. Data mining: concepts and techniques. Elsevier; 2011.

[14] Cunningham P, Delany SJ. k-Nearest neighbour classifiers. Mult Classif Syst. Springer; 2007;34: 1–17.

[15] Adeniyi DA, Wei Z, Yongquan Y. Automated web usage data mining and recommendation system using K-Nearest Neighbor (KNN) classification method. Appl Comput Informatics. 2016;12: 90–108. 10.1016/j.aci.2014.10.001

[16] Bidder OR, Campbell HA, Gómez-Laich A, Urgé P, Walker J, Cai Y, et al Love Thy Neighbour: Automatic Animal Behavioural Classification of Acceleration Data Using the K-Nearest Neighbour Algorithm. Kalueff A V., editor. PLoS One. Public Library of Science; 2014;9: e88609 10.1371/journal.pone.0088609 24586354 PMC3931648

[17] Holmström H, Nilsson M, Ståhl G. Simultaneous Estimations of Forest Parameters using Aerial Photograph Interpreted Data and the *k* Nearest Neighbour Method. Scand J For Res. Taylor & Francis Group; 2001;16: 67–78. 10.1080/028275801300004424

[18] Horton P, Nakai K. Better prediction of protein cellular localization sites with the k nearest neighbors classifier. Proceedings Int Conf Intell Syst Mol Biol. 1997;5: 147–52. Available: http://www.ncbi.nlm.nih.gov/pubmed/9322029 9322029

[19] Mehrotra R, Sharma A. Conditional resampling of hydrologic time series using multiple predictor variables: A K-nearest neighbour approach. Adv Water Resour. 2006;29: 987–999. 10.1016/j.advwatres.2005.08.007

[20] Noguchi T, Demura S, Takahashi K. Relationships between sit-ups and abdominal flexion strength tests and the thickness of each abdominal muscle. Adv Phys Educ. Scientific Research Publishing; 2013;3: 84.

[21] Fleishman EA. The structure and measurement of physical fitness. Prentice-Hall; 1964;

[22] Taha Z, Musa RM, Abdul Majeed A P.P., Alim MM, Abdullah MR. The identification of high potential archers based on fitness and motor ability variables: A Support Vector Machine approach. Hum Mov Sci. 2018;57: 184–193. 10.1016/j.humov.2017.12.008 29248809

[23] Vach W, Roßner R, Schumacher M. Neural networks and logistic regression: Part II. Comput Stat Data Anal. North-Holland; 1996;21: 683–701. 10.1016/0167-9473(95)00033-X

[24] Schumacher M, Roßner R, Vach W. Neural networks and logistic regression: Part I. Comput Stat Data Anal. North-Holland; 1996;21: 661–682. 10.1016/0167-9473(95)00032-1

[25] Lemon SC, Roy J, Clark MA, Friedmann PD, Rakowski W. Classification and Regression Tree Analysis in Public Health: Methodological Review and Comparison With Logistic Regression. Ann Behav Med. 2003;26: 172–181. Available: https://link.springer.com/content/pdf/10.1207%2FS15324796ABM2603_02.pdf 10.1207/S15324796ABM2603_02 14644693

[26] Worth AP, Cronin MT. The use of discriminant analysis, logistic regression and classification tree analysis in the development of classification models for human health effects. J Mol Struct THEOCHEM. Elsevier; 2003;622: 97–111. 10.1016/S0166-1280(02)00622-X

[27] Landers DM, Petruzzello SJ, Salazar W, Crews DJ, Kubitz KA, Gannon TL, et al The influence of electrocortical biofeedback on performance in pre-elite archers. Med Sci Sport Exerc. Lippincott Williams & Wilkins; 1991; 1997806

[28] Keast D, Elliott B. Fine body movements and the cardiac cycle in archery. J Sports Sci. Taylor & Francis Group; 1990;8: 203–213. 10.1080/02640419008732146 2084267

[29] Calvert TW, Banister EW, Savage M V, Bach T. A systems model of the effects of training on physical performance. IEEE Trans Syst Man Cybern. IEEE; 1976; 94–102.

[30] Elferink-Gemser MT, Visscher C, Lemmink KAPM, Mulder T. Multidimensional performance characteristics and standard of performance in talented youth field hockey players: A longitudinal study. J Sports Sci. Taylor & Francis; 2007;25: 481–489. 10.1080/02640410600719945 17365535

