# Supplementary material for: A machine learning approach of predicting high potential archers by means of physical fitness indicators
Source: PLoS One. 2019 Jan 3;14(1):e0209638. doi: 10.1371/journal.pone.0209638 (PMC6317817; doi:10.1371/journal.pone.0209638)
Supplement: S1 File — (DOCX) [file pone.0209638.s001.docx]

**Classification assessment formulae**

The confusion matrix of a two-class classification problem is shown in Table A1.

**Table A1. Confusion Matrix**

|  | Predicted class | |
| --- | --- | --- |
| Actual Class | a | b |
|  | c | d |

where a, b, c and d represents the true positives (the number of positive samples correctly predicted), false negatives (number of positive samples predicted as negative), false positives (negative samples predicted as positive) and true negatives (the number of negative samples predicted correctly). The formulae of the classifier performance assessment parameters are formulated as follows:

$ACC=\frac{a+d}{a+b+c+d}$ (1)

$SENS=\frac{a}{a+b}$ (2)

$SPEC=\frac{d}{d+c}$ (3)

$PREC=\frac{a}{a+c}$ (4)

$ERR==\frac{c+b}{a+b+c+d}$ (5)

$MCC=\frac{\left(a*c\right)+\left(b*d\right)}{\left(a+b\right)+\left(a+d\right)+\left(c+b\right)+\left(c+d\right)}$ (6)
